# Geometric imaging of borophene polymorphs with functionalized probes

**DOI:** 10.1038/s41467-019-09686-w

**Published:** 2019-04-09

**Authors:** Xiaolong Liu, Luqing Wang, Shaowei Li, Matthew S. Rahn, Boris I. Yakobson, Mark C. Hersam

**Affiliations:** 1 Applied Physics Graduate Program, Northwestern University, Evanston, IL 60208 USA; 2 Department of Materials Science and NanoEngineering, Rice University, Houston, TX 77005 USA; 3 Department of Materials Science and Engineering, Northwestern University, Evanston, IL 60208 USA; 4 Department of Chemistry, Rice University, Houston, TX 77005 USA; 5 Department of Chemistry, Northwestern University, Evanston, IL 60208 USA; 6 Department of Electrical Engineering and Computer Science, Northwestern University, Evanston, IL 60208 USA

## Abstract

A common characteristic of borophene polymorphs is the presence of hollow hexagons (HHs) in an otherwise triangular lattice. The vast number of possible HH arrangements underlies the polymorphic nature of borophene, and necessitates direct HH imaging to definitively identify its atomic structure. While borophene has been imaged with scanning tunneling microscopy using conventional metal probes, the convolution of topographic and electronic features hinders unambiguous identification of the atomic lattice. Here, we overcome these limitations by employing CO-functionalized atomic force microscopy to visualize structures corresponding to boron-boron covalent bonds. Additionally, we show that CO-functionalized scanning tunneling microscopy is an equivalent and more accessible technique for HH imaging, confirming the *v*_1/5_ and *v*_1/6_ borophene models as unifying structures for all observed phases. Using this methodology, a borophene phase diagram is assembled, including a transition from rotationally commensurate to incommensurate phases at high growth temperatures, thus corroborating the chemically discrete nature of borophene.

## Abstract (web summary)

Borophene, or 2D boron, is highly polymorphic with many predicted lattice arrangements, complicating the identification of its atomic structure. Here, the authors use functionalized-tip scanning probe microscopy to directly resolve the atomic lattice structures of several borophene polymorphs.

## Introduction

The recent experimental realization of 2D boron^1–8^ (i.e., borophene) has spurred broad interest in its unique material attributes such as in-plane anisotropy^9^, massless Dirac fermions^10^, and seamless phase intermixing^7^. The polymorphic nature of borophene^2,3^, which is rooted in the rich bonding configurations among boron atoms, further distinguishes it from other 2D materials and offers an additional means for tailoring its material properties. However, when combined with the large number of predicted lattice structures for borophene, polymorphism introduces challenges for the unambiguous identification of borophene atomic structures experimentally. For example, while conventional metal-tip scanning tunneling microscopy (STM) has provided atomic-scale characterization of borophene^4–7^, the convolution of topographic and electronic structure in the resulting images coupled with Moiré superlattices with the underlying substrate leads to experimental uncertainties that do not provide sufficient differentiation among possible borophene lattices. While comparison of available experimental data with density functional theory calculations and simulated images help exclude some possibilities, sufficient ambiguity remains such that the literature lacks consensus for many observed borophene phases^5,11^. Consequently, an experimental method for definitively identifying borophene atomic structures is needed in order to establish the fundamental structure–property relationships that underlie emerging borophene applications. Since the first evidence of enhanced scanning probe spatial resolution with pentacene-functionalized tips^12^, deliberate tip functionalization with CO^13^, H_2_^14,15^, Xe^16^, and CuO_x_^17,18^ has been utilized to resolve spatial details down to the subatomic regime. Although the physical mechanisms that lead to this improved spatial resolution is still under debate^19^, geometric imaging with functionalized tips reveals inter-molecular and intra-molecular chemical bonds, particularly in carbon-based molecular or graphitic systems^20–22^.

In an extension of this methodology, we show here that cryogenic ultrahigh vacuum non-contact CO-functionalized atomic force microscopy (CO-AFM) geometrically resolves features that are consistent with boron-boron covalent bonds. We further demonstrate that CO-functionalized STM (CO-STM)^22^ resolves borophene HHs and thus provides equivalently unambiguous geometric identification of borophene atomic lattice structures in a manner that is experimentally less demanding. By applying this approach to several borophene phases produced at different growth temperatures, a borophene phase diagram is constructed that includes multiple borophene polymorphs with different rotational alignments on Ag(111). In particular, a transition from rotationally commensurate to rotationally incommensurate borophene phases is observed at high growth temperatures, which suggests that borophene is a chemically discrete two-dimensional material as opposed to a surface reconstruction or alloy with the underlying Ag(111) growth substrate.

## Results

### Non-rotated borophene phases

**Fig. 1:**
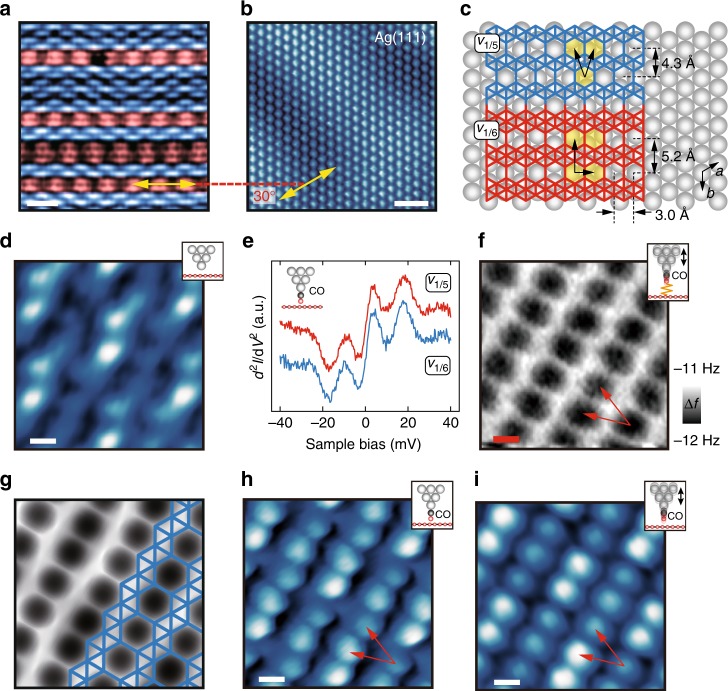
Imaging *v*_1/5_ phase borophene with bare and functionalized probes. **a** Bare-tip STM image of intermixed *v*_1/5_ (colored blue) and *v*_1/6_ (colored red) phase borophene. **b** A neighboring Ag(111) surface, where the angle between the HH rows of borophene (horizontal) in **a** and the Ag atomic chains is 30° as expected. **c** Schematic illustration of the rotational orientation of *v*_1/5_ and *v*_1/6_ phase borophene on Ag(111). The arrows mark the unit cells of the HH lattices in each phase. **d** Bare-tip STM image of *v*_1/5_ phase borophene. **e** Inelastic electron tunneling spectroscopy on borophene with a CO-functionalized tip. **f** CO-AFM image of *v*_1/5_ phase borophene. **g** Simulated CO-AFM image of *v*_1/5_ phase borophene with overlaid atomic structure. **h** CO-STM and **i** dynamic CO-STM image of *v*_1/5_ phase borophene. The red arrows in **f**, **h**, and **i** denote the unit cells corresponding to the HH lattice. *V*_s_ = −10 mV in **a**, 20 mV in **b**, 100 mV in **d**, −7 mV in **h**, and 15 mV in **i**. Scale bars, 2 nm in **a**, 1 nm in **b**, 2 Å in **d**, **f**, **h**, **i**

Borophene polymorphs are characterized by different arrangements and concentrations of HHs in an otherwise 2D triangular lattice. Figure 1a shows a conventional bare-tip STM image of intermixed *v*_1/5_ (colored blue) and *v*_1/6_ (colored red) phase borophene grown at a previously established temperature of ~450 °C^7^. By comparing these borophene structures with the atomic lattice of the Ag(111) growth substrate (Fig. 1b), the horizontal HH row directions of both phases are found to be 30° rotated with respect to the Ag atomic chains (directions ***a*** or ***b*** in Fig. 1c), consistent with previously proposed structural models^4^ as shown in Fig. 1c. For *v*_1/5_ and *v*_1/6_ phase borophene, the HHs are arranged in a staggered and aligned manner, respectively, with the unit cells of the HH lattices indicated by the black arrows. High-resolution STM imaging of *v*_1/5_ phase borophene with a bare metal tip (Fig. 1d) reveals a strong brick-wall type Moiré pattern modulating finer features.

In an effort to gain higher spatial resolution, imaging of these phases was repeated with CO-AFM. In particular, Figure 1e shows the characteristic inelastic electron tunneling spectra of CO obtained on borophene after decorating the metal tips with a CO molecule (hindered translation and rotation modes at 3.2 meV and 17.6 meV, respectively), confirming successful tip functionalization (Supplementary Fig. 1)^23^. In contrast to the STM image in Fig. 1d, the constant height CO-AFM image in Fig. 1f geometrically resolves the HH lattice and verifies the proposed *v*_1/5_ phase borophene structure, where the HHs are separated by bright ridges at positions expected for boron–boron covalent bonds. In agreement with the experimental image, Figure 1g shows a simulated CO-AFM image^24,25^ with an overlaid *v*_1/5_ phase atomic structure. While the apparent bonding structures of the 6-membered boron rings (i.e., HHs) are clearly resolved, minimal contrast is observed for the triangular boron lattice, where only 3-membered boron rings exist. This limited spatial resolution is reasonable given the fact that 4-membered carbon rings have also not been resolved with CO-AFM^26^. Nevertheless, resolving the HH lattice is sufficient for determining the structures of borophene polymorphs and thus CO-AFM is a definitive tool for identifying borophene atomic lattice structures^3^.

In light of the highly demanding nature of non-contact AFM in terms of instrumentation and operation, we further demonstrate that more experimentally accessible CO-STM imaging provides equivalent geometric information in Fig. 1h. Compared to the bare-tip STM image in Fig. 1d, a lattice of staggered protrusions (red arrows) matching the HH lattice are clearly resolved in addition to the less-obvious Moiré pattern. By performing CO-STM imaging in dynamic mode^27^ (i.e., with an oscillating STM tip), this contrast is further enhanced (Fig. 1i) at milder imaging conditions (Supplementary Fig. 2), which helps improve the stability of the adsorbed CO molecule on the tip apex.

### 30°-rotated borophene phases

**Fig. 2:**
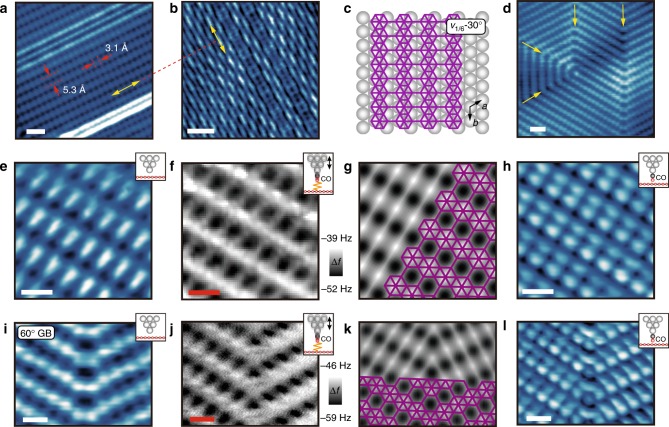
Imaging *v*_1/6_−30° phase borophene structures with bare and functionalized probes. Bare-tip STM images of **a** a *v*_1/6_−30° phase borophene domain and **b** a neighboring *v*_1/5_ phase borophene, where the HH rows in each phase are perpendicular (yellow arrows). **c** Schematic of *v*_1/6_−30° phase borophene. **d** Bare-tip STM image of polycrystalline *v*_1/6_−30° phase borophene with multiple 60° GBs (yellow arrows). **e** Bare-tip, **f** CO-AFM, **g** simulated CO-AFM, and **h** CO-STM images of *v*_1/6_−30° phase borophene. The atomic structure is overlaid in **g**. **i** Bare-tip, **j** CO-AFM, **k** simulated CO-AFM, and **l** CO-STM images of a 60° GB within *v*_1/6_−30° phase borophene. The atomic structure is overlaid in **k**. *V*_s_ = −3 mV in **a**, 4 mV in **b**, −34 mV in **d**, 9 mV in **e**, 4 mV in **h**, −34 mV in **i**, and 4 mV in **l**. Scale bars, 1 nm in **a**, 2 nm in **b**, 1 nm in **d**, 5 Å in **e**, **f**, **h**–**j**, **l**

At higher growth temperatures (~500 °C), *v*_1/5_ phase borophene dominates^7^, although another phase with a rectangular lattice also begins to appear (Fig. 2a). This structure was first observed in high-temperature growth and assigned to a buckled triangular lattice^5^. The 3 Å × 5 Å lattice constants are also indicative of *v*_1/6_ phase borophene, which normally grows at lower temperatures^4^. However, the absence of a linear Moiré pattern typically seen for *v*_1/6_ phase borophene suggests that this phase might be the *v*_1/6_ sheet on Ag(111) with a different orientation with respect to the underlying Ag(111) lattice as suggested previously^28,29^. Indeed, instead of being parallel as in the low-temperature *v*_1/6_ phase (Fig. 1c), the HH rows in this case are perpendicular to those of a neighboring *v*_1/5_ borophene domain in Fig. 2b (yellow arrows). We thus label this phase as *v*_1/6_ −30° as schematically shown in Fig. 2c, indicating that the borophene lattice orientation is rotated by 30° on Ag(111) compared to the low-temperature *v*_1/6_ phase.

A polycrystalline domain of the *v*_1/6_−30° phase with 60° grain boundaries (GBs; yellow arrows) is shown in Fig. 2d. Figure 2e, f are high-resolution STM and CO-AFM images of *v*_1/6_−30° phase borophene, respectively, where the aligned HHs are clearly resolved in Fig. 2f with neighboring HHs separated by bright ridges at positions expected for boron–boron covalent bonds. In Fig. 2g, the simulated CO-AFM image with an overlaid atomic structure again agrees well with experimental observations, confirming our structural assignment as *v*_1/6_−30°. On the other hand, simulated CO-AFM images of a buckled triangular lattice deviate from the experimental image (Supplementary Fig. 3). Compared to the bare tip STM image, each bright protrusion splits into two protrusions with inequivalent brightness in the CO-STM image in Fig. 2h. A series of bare-tip STM, CO-AFM, and CO-STM images of a 60° GB in *v*_1/6_−30° phase borophene is shown in Fig. 2i, j, l, respectively. Based on the apparent bonding positions resolved in Fig. 2j, we propose an atomic structure of the GB seamlessly connecting adjacent grains (Fig. 2k). This proposed structure yields a simulated CO-AFM image (Fig. 2k) that agrees well with experimental observation, further supporting our assignment of the *v*_1/6_−30° phase.

**Fig. 3:**
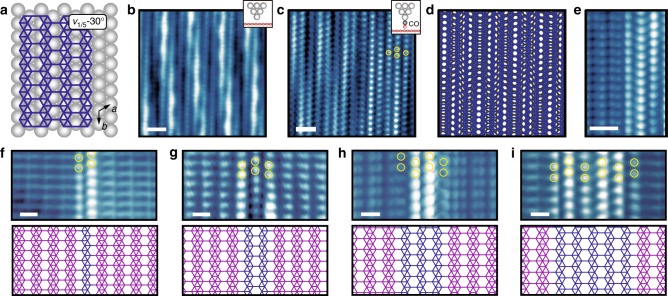
Resolving *v*_1/5_−30° phase borophene and its intermixing with the *v*_1/6_−30° phase. **a** Schematic of *v*_1/5_−30° phase borophene. **b** Bare-tip, and **c** CO-STM images of undulating *v*_1/5_−30° phase borophene. The yellow circles in **c** indicate the staggered HH pattern. **d** Schematic of the undulations resulting from a lattice mismatch between borophene and Ag(111). **e** CO-STM image of the phase boundary between *v*_1/6_−30° (left) and *v*_1/5_−30° phase borophene. **f**–**i** CO-STM images of *v*_1/5_−30° line defects in *v*_1/6_−30° phase borophene with different widths. The yellow circles denote the staggered arrangement of the HH patterns, with the corresponding structure schematics provided in the bottom row. *V*_s_ = −3 mV in **b**, **c**, −7 mV in **e**, **f**, −1 mV in **g**, 4 mV in **h**, and −7 mV in **i**. Scale bars, 1 nm in **b**, **c**, **e**, 5 Å in **f**–**i**

As established previously, intermixing of *v*_1/5_ and *v*_1/6_ phase borophene takes place when the two phases have rotationally aligned HH rows^7^. Therefore, it is reasonable to expect that *v*_1/5_−30° phase borophene (schematically shown in Fig. 3a) would intermix with the *v*_1/6_−30° phase at appropriate growth conditions. Indeed, at a higher growth temperature (~550 °C), an undulated structure appears as shown in the bare-tip STM image in Fig. 3b, which has previously been referred to as the striped phase^5^ and suggested to be induced by an undulating Ag surface reconstruction under a *v*_1/6_−30° sheet^28^. However, based on the CO-STM image in Fig. 3c, we conclude that this phase adopts the *v*_1/5_ borophene structure as evidenced by the staggered arrangement of protrusions (yellow circles) representing the HH lattice. Furthermore, instead of the brick-wall type Moiré pattern observed for the low-temperature *v*_1/5_ phase, the apparent larger-scale undulation results from a ~4% larger inter-HH spacing along the HH rows than the interatomic spacing of the underlying Ag, which is reproduced qualitatively in Fig. 3d by rotating *v*_1/5_ phase borophene by 30° on Ag(111). The coexistence of this structure with *v*_1/6_−30° phase borophene with parallel HH rows and a seamless phase boundary is shown in the CO-STM image in Fig. 3e, confirming a rotation angle of 30° and thus the identity of this phase as *v*_1/5_−30°. Since phase intermixing results from line defects in each phase adopting the structure of the other phase^7^, we identify various *v*_1/5_−30° line defects in *v*_1/6_−30° phase borophene with different widths as shown in the top row of Fig. 3f–i (CO-STM images). The yellow circles denote the staggered HH pattern in the *v*_1/5_−30° regions, while the corresponding structures are displayed in the bottom row. Additional CO-STM and CO-AFM images of intermixed *v*_1/6_−30° and *v*_1/5_−30° phase borophene are provided in Supplementary Fig. 4.

### Rotationally incommensurate borophene phases

**Fig. 4:**
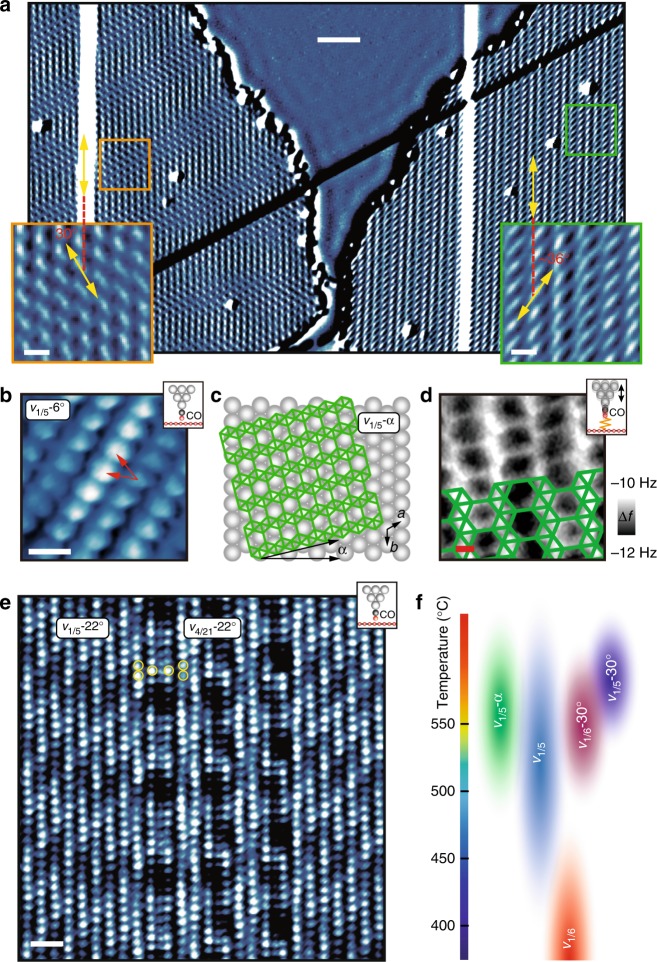
Imaging rotationally incommensurate phases of borophene with functionalized probes. **a** Large-scale derivative image of the bare-tip STM topography for *v*_1/5_ (left) and *v*_1/5_−6° (right) phase borophene domains. The insets are the zoomed-in images in the orange and green squares. The relative orientations of the HH rows in each phase with respect to the Ag atomic chains (vertical direction) are labeled. **b** CO-STM image of *v*_1/5_−6° phase borophene, where the red arrows indicate the staggered HH lattice pattern. **c** Schematic of rotationally incommensurate borophene phases, where *α* denotes the angle by which the sheet is rotated from *v*_1/5_ phase borophene. **d** CO-AFM image of *v*_1/5_−6° phase borophene with overlaid atomic structure. **e** CO-STM image of *v*_1/5_−22° phase borophene with self-assembled *v*_1/6_−22° line defects. The yellow circles denote the aligned and staggered HH patterns. **f** Phase diagram of borophene with respect to growth temperature. Individual ovals represent each phase. *V*_s_ = 190 mV in **a**, −2 mV in **b**, and 22 mV in **e**. Scale bars, 5 nm in **a**, 1 nm in the insets in **a**, 5 Å in **b**, 2 Å in **d**, 1 nm in **e**

All of the borophene phases discussed thus far (i.e., *v*_1/6_, *v*_1/5_, *v*_1/6_−30°, and *v*_1/5_−30°) are reasonably characterized as being rotationally commensurate with the underlying growth substrate since the HH rows are aligned along high symmetry directions of the Ag(111) surface. In contrast, at similar conditions where phase intermixing is observed for *v*_1/6_−30° and *v*_1/5_−30° borophene, another new structure is observed as shown in the derivative image of the bare-tip STM topography in Fig. 4a. As seen in the zoomed-in inset images, the borophene domain on the left is *v*_1/5_ phase borophene with a characteristic brick-wall type Moiré pattern (orange square), whereas the domain on the right shows a distorted brick-wall type Moiré pattern (green square). The CO-STM image of this new domain structure is provided in Fig. 4b and reveals a HH lattice with staggered protrusions (red arrows) that are indicative of a *v*_1/5_ borophene sheet. However, due to the ~36° angle (instead of 30° angle for *v*_1/5_) between the directions of the HH rows and the Ag atomic chains (vertical direction, Fig. 4a), this borophene sheet is denoted as *v*_1/5_−6°. Rotationally incommensurate *v*_1/5_ borophene sheets are generally depicted in Fig. 4c as *v*_1/5_-*α*, where *α* is the rotation angle by which the sheet deviates from the *v*_1/5_ phase. In Fig. 4d, a CO-AFM image of the *v*_1/5_−6° phase with an overlaid atomic structure confirms the assignment as a *v*_1/5_ sheet.

In addition to *α* = 6°, other rotationally incommensurate phases such as *v*_1/5_−9° and *v*_1/5_−22° phase borophene have been observed (Supplementary Fig. 5). As an example, the CO-STM image in Fig. 4e resolves the HH lattice of *v*_1/5_−22° phase borophene with periodically self-assembled *v*_1/6_−22° line defects in the middle, as evidenced by the staggered and aligned HH patterns indicated by the yellow circles, respectively. Equivalently, the self-assembled periodic line defects can be categorized as *v*_4/21_−22° phase borophene (another example is shown in Supplementary Fig. 5) in a manner analogous to previously reported periodic intermixed phases for non-rotated borophene growth (i.e., *α* = 0°)^7^. Combining all of the observations discussed above and established previously, a phase diagram for borophene growth with respect to temperature is summarized in Fig. 4f, where each phase is represented by an oval. The overall trend is that borophene growth transitions from rotationally commensurate phases to rotationally incommensurate phases at high temperatures. Significantly, the existence of rotationally incommensurate phases provides corroborating evidence that borophene layers are chemically discrete from the underlying Ag surface^8^. It should be further noted that the range of observed borophene phases show similar electronic properties (Supplementary Fig. 6) with no measurable differences in lattice constants.

In summary, we have utilized CO-functionalized scanning probe microscopy to geometrically image and determine the atomic lattice structures of various borophene polymorphs. In addition to CO-AFM, we establish CO-STM as an alternative and comparatively more accessible technique for unambiguously determining borophene atomic structures. Using these methods, we assigned structure models for several phases of borophene in addition to resolving features consistent with boron–boron covalent bonds. In all cases, the borophene phases are found to consist of *v*_1/5_ and/or *v*_1/6_ domains, although the orientation of these domains with respect to the underlying Ag(111) substrate transitions from rotationally commensurate to rotationally incommensurate at the highest growth temperatures. The resulting phase diagram explains all observations of borophene to date with the newest rotationally incommensurate phases providing strong evidence that borophene is a chemically discrete two-dimensional material as opposed to a surface reconstruction or alloy with the underlying Ag(111) growth substrate. This conclusion is further supported by the recent synthesis of borophene on Cu(111)^30^. The ability of CO-AFM and CO-STM to resolve the complicated phase diagram for borophene polymorphs suggests that CO-functionalized probes can be similarly employed for the unambiguous determination of atomic structures for other emerging synthetic 2D materials and their heterostructures^31^.

## Methods

### Borophene growth

Borophene growth is described in detail in an earlier report^7^. Briefly, a solid boron rod (ESPI metals, 99.9999% purity) is evaporated onto Ag(111) films (~600 nm thick) on mica (Princeton Scientific Corp.) in an ultrahigh vacuum preparation chamber (~1 × 10^−10^ mbar) with an electron-beam evaporator (FOCUS) for a duration of ~30 min to achieve submonolayer coverage. The clean Ag(111) surface for borophene growth is prepared by repeated 30 min Ar ion sputtering at 1 × 10^−5^ mbar followed by 30 min annealing at 550 °C.

### Scanning probe microscopy and tip-functionalization

Low temperature STM/STS and non-contact AFM characterization is performed on a Scienta Omicron LT STM (~2 × 10^−11^ mbar) at ~4 K interfaced with Nanonis (SPECS) control electronics using qPlus AFM sensors (mounted W tips from Scienta Omicron). Tip functionalization is achieved by leaking CO molecules into the STM chamber (1 × 10^−7^ mbar for 40 s) and picking up individual CO molecules by ramping the sample bias down to −2 mV and the tunneling current up to 1 nA. For AFM measurements, an oscillation amplitude of 1–2 Å is used. AFM image simulation is based on the method developed by Hapala and coworkers^24,25^. A lock-in amplifier (SRS model SR850) is used for STS measurements with 2 mV_RMS_ amplitude and ~0.8 kHz modulation frequency. Gwyddion software is used for image processing.

## Supplementary information

Supplementary Information

## Data Availability

All data needed to evaluate the conclusions in the paper are present in the paper and/or the Supplementary Information. Additional data related to this paper may be requested from the authors.
