# Supplementary material for: Geometric imaging of borophene polymorphs with functionalized probes
Source: Nat Commun. 2019 Apr 9;10:1642. doi: 10.1038/s41467-019-09686-w (PMC6456592; doi:10.1038/s41467-019-09686-w)
Supplement: Supplementary file 1 — Supplementary Information [file 41467_2019_9686_MOESM1_ESM.pdf]

Supplementary Information

Geometric imaging of borophene polymorphs with functionalized probes

*Xiaolong Liu[1], Luqing Wang[2], Shaowei Li[3], Matthew S. Rahn[3], Boris I. Yakobson[2,4], and Mark C. Hersam[1,3,5,6]**

[1]Applied Physics Graduate Program, Northwestern University, Evanston, IL 60208, USA

[2]Department of Materials Science and NanoEngineering, Rice University, Houston, TX 77005, USA

[3]Department of Materials Science and Engineering, Northwestern University, Evanston, IL 60208, USA

[4]Department of Chemistry, Rice University, Houston, TX 77005, USA

[5]Department of Chemistry, Northwestern University, Evanston, IL 60208, USA

[6]Department of Electrical Engineering and Computer Science, Northwestern University, Evanston, IL 60208, USA

*Correspondence should be addressed to: m-hersam@northwestern.edu

Table of Contents

- **Supplementary Fig. 1.** Tip-functionalization with CO molecules.
- **Supplementary Fig. 2.** Milder imaging conditions with dynamic CO-functionalized scanning tunneling microscopy (CO-STM).
- **Supplementary Fig. 3.** Additional simulated non-contact CO-functionalized atomic force microscopy (CO-AFM) images.
- **Supplementary Fig. 4.** Additional images of $\nu_{1/6}$-30º and $\nu_{1/5}$-30º phase borophene.
- **Supplementary Fig. 5.** Additional images of $\nu_{1/5}$-22º, $\nu_{4/21}$-22º, and $\nu_{1/5}$-9º phase borophene.
- **Supplementary Fig. 6.** Scanning tunneling spectroscopy (STS) measurements of different borophene phases.

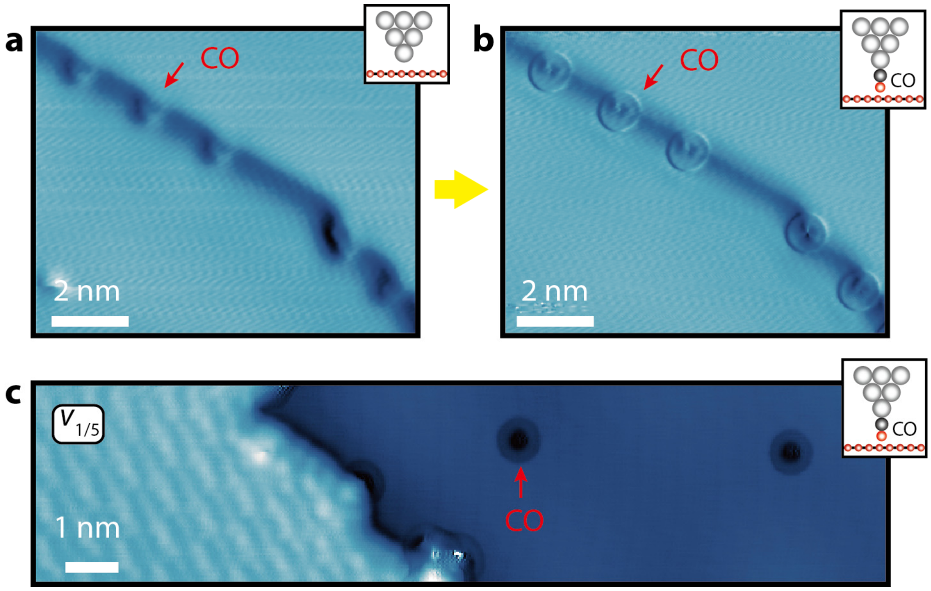

Supplementary Figure 1. Tip-functionalization with CO molecules. **a**,**b**, Derivative images of STM topography of CO molecules adsorbed on a Ag(111) step edge with a (**a**) bare tip, and (**b**) a CO-tip. **c**, CO-STM image of a $v_{1/5}$ borophene domain next to a Ag terrace. The appearance of the CO molecules on the Ag terrace confirms the tip as CO-functionalized. $V_s = 30$ mV in **a** and **b**, and 10 mV in **c**.

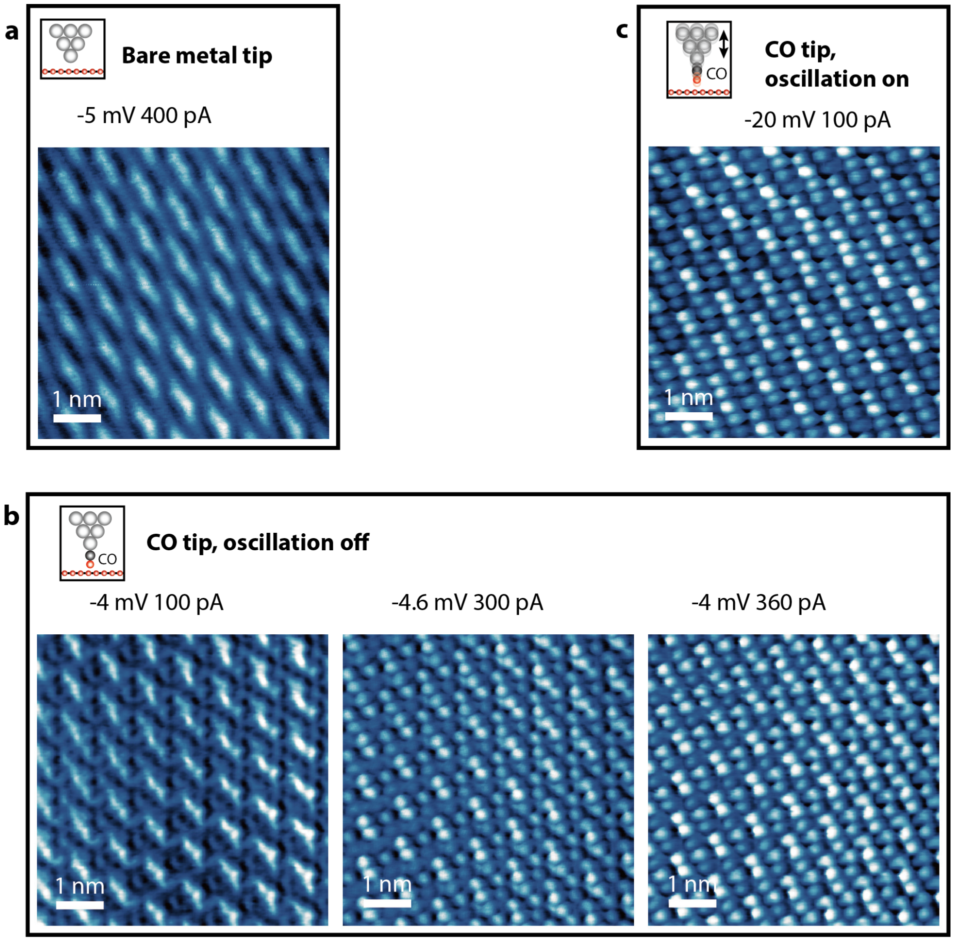

Supplementary Figure 2. Milder imaging conditions with dynamic CO-STM. **a**, A typical STM image of $v_{1/5}$ phase borophene with a bare metal tip (tungsten). **b**, A series of CO-STM images of the same region with increasing tunneling current, where the HH lattice is most clearly resolved at the highest tunneling currents. **c**, Dynamic CO-STM image of the same region (~1 nm tip oscillation). Evidently, dynamic CO-STM imaging under milder imaging conditions (i.e., lower tunneling currents) yields comparable resolution of the HH lattice compared to standard CO-STM imaging at more perturbative imaging conditions (i.e., higher tunneling currents). The net effect is that dynamic CO-STM leads to enhanced stability of the CO molecule on the tip apex, especially under conditions that yield the highest spatial resolution.

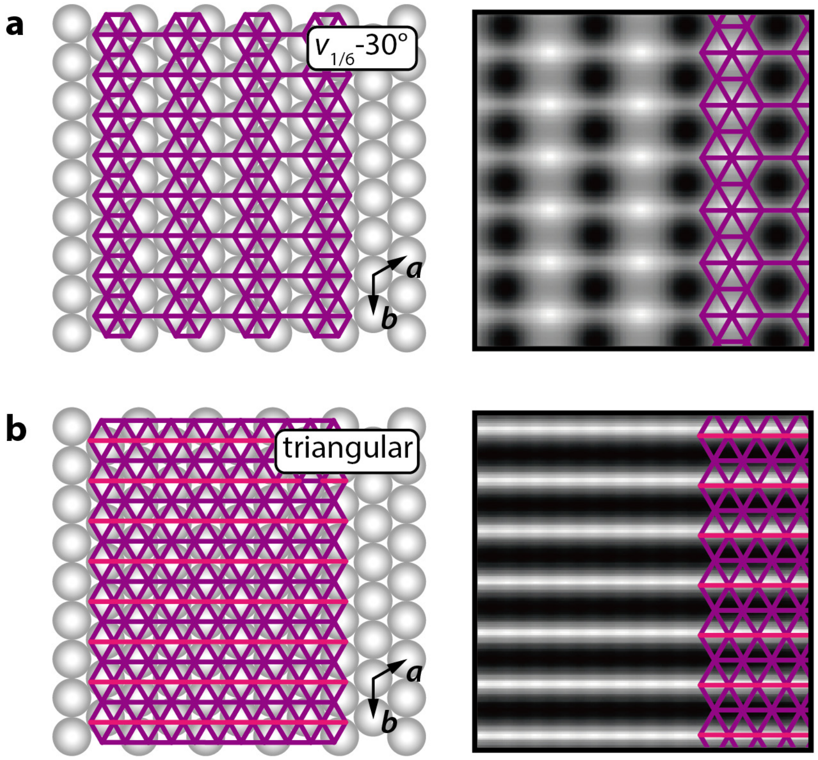

Supplementary Figure 3. Additional simulated CO-AFM images. **a**,**b**, Schematics and simulated CO-AFM images of (**a**) $v_{1/6}$-30º phase borophene and (**b**) triangular lattice borophene (the brighter pink lines indicate upward buckling). The simulated image of the triangular lattice significantly deviates from the experimental observation in Figure 2f.

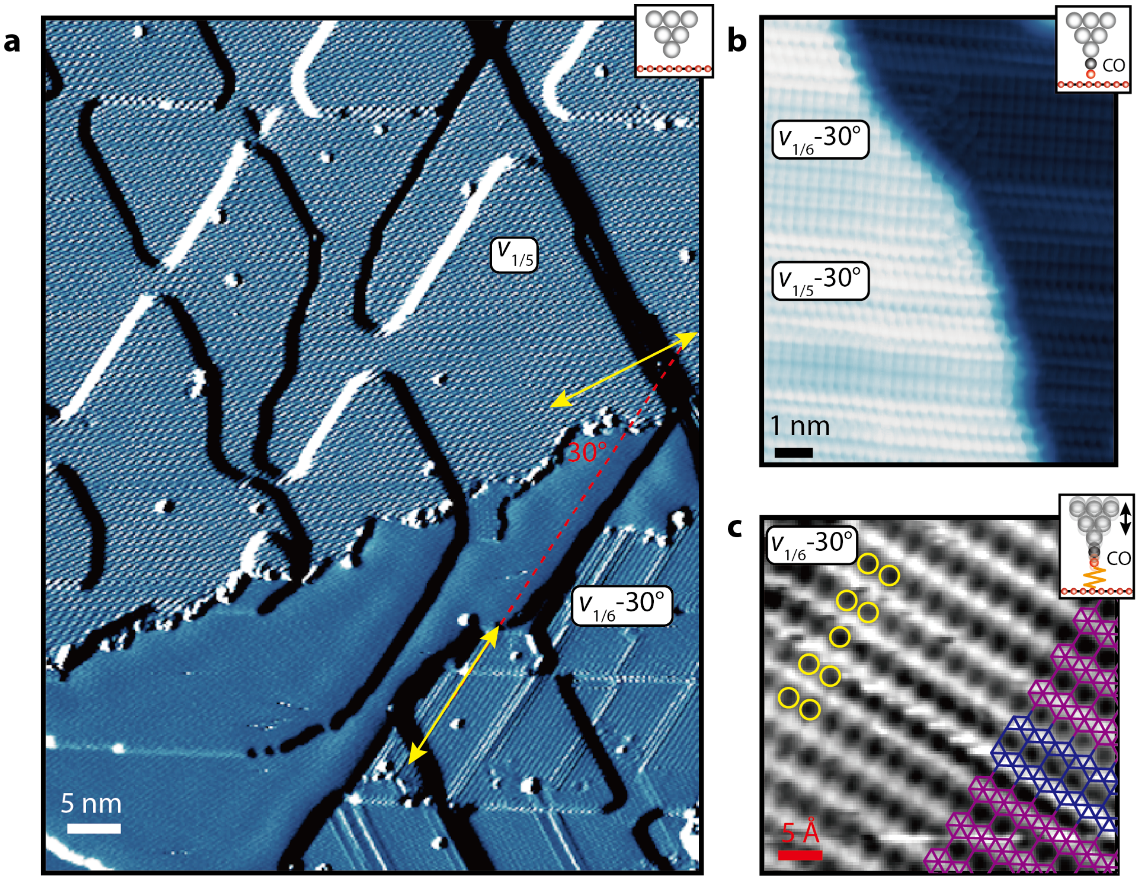

Supplementary Figure 4. Additional images of $v_{1/6}$-30º and $v_{1/5}$-30º phase borophene. **a**, Large-scale derivative image of bare-tip STM topography containing $v_{1/5}$ and $v_{1/6}$-30º phase borophene domains, which allows the relative orientations to be directly measured. In this case, the angle between the HH rows (yellow arrows) is 30º. **b**, CO-STM image of intermixed $v_{1/6}$-30º and $v_{1/5}$-30º borophene phases. **c**, CO-AFM image of a $v_{1/5}$-30º-structured line defect in $v_{1/6}$-30º phase borophene with an overlaid structure model. The staggered and aligned arrangements of the HHs in each phase are indicated by the yellow circles. V_s = -100 mV in **a** and -7 mV in **b**.

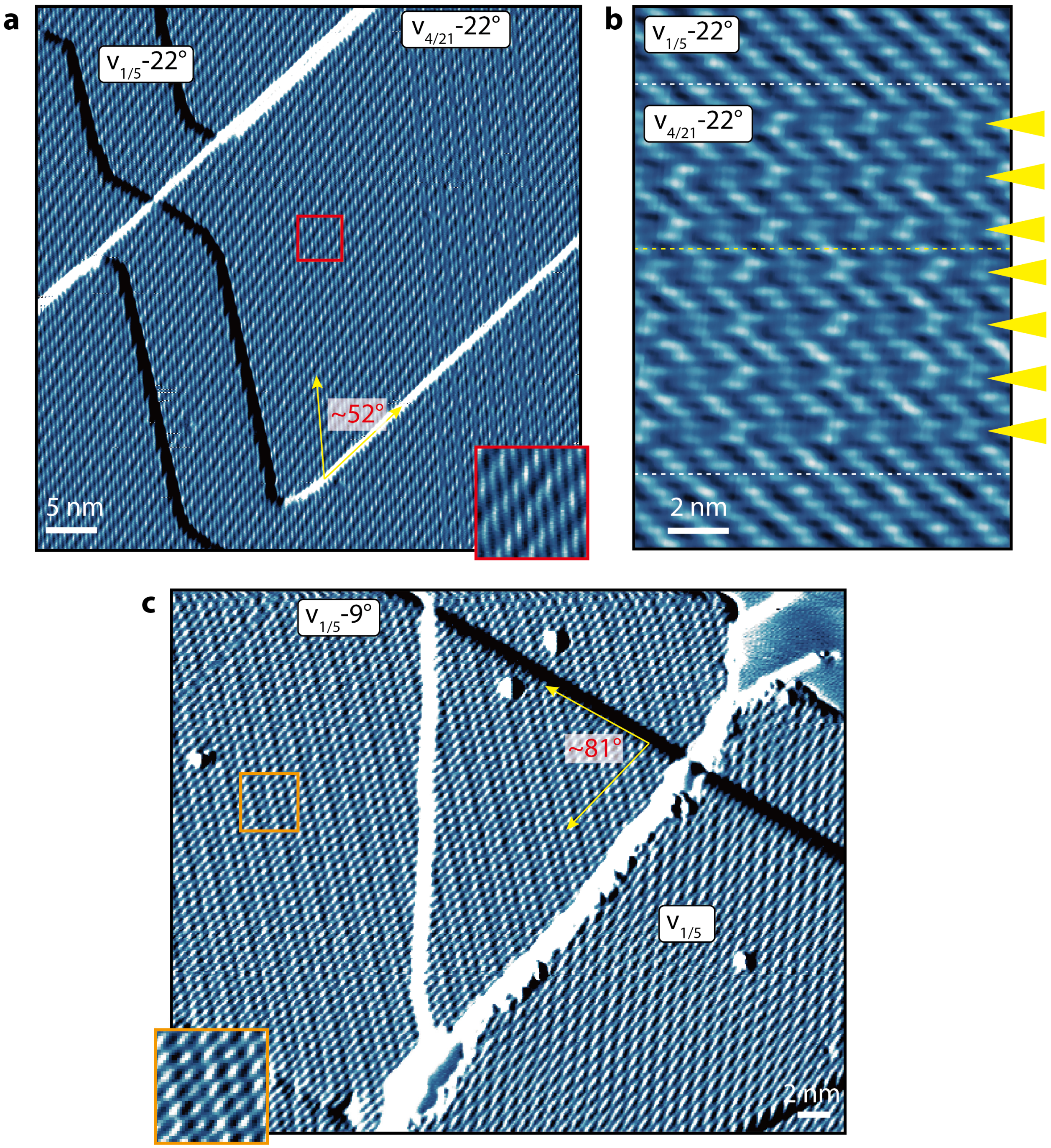

Supplementary Figure 5. Additional images of $v_{1/5}$-22°, $v_{4/21}$-22°, and $v_{1/5}$-9° phase borophene. **a**, Large-scale derivative image of bare-tip STM topography of a $v_{1/5}$-22° phase borophene domain with a $v_{4/21}$-22° phase on the right. Since the HH row direction is ~52° rotated from the Ag atomic chain direction (yellow arrows), the rotation angle α with respect to $v_{1/5}$ is 22°. The inset shows

the zoomed-in image of the region in the red square. **b**, Zoom-in derivative image of bare-tip STM topography of the $v_{4/21}$-22º domain shown in (**a**). The yellow arrow heads indicate positions of the periodic $v_{1/6}$-22º line defects in $v_{1/5}$-22º phase borophene, forming equivalently the $v_{4/21}$-22º phase. **c**, Large-scale derivative image of bare-tip STM topography of $v_{1/5}$-9º phase borophene neighboring $v_{1/5}$ phase borophene. The inset shows the zoomed-in image of the region in the orange square. While the HH rows of the $v_{1/5}$ phase borophene are perpendicular (or 30º rotated) to the Ag atomic chains, the angle between the HH rows of $v_{1/5}$-9º phase borophene and the Ag atomic chains is ~81º (yellow arrows). Therefore, the phase is 9º rotated from the $v_{1/5}$ phase and is denoted as $v_{1/5}$-9º. V_s = 15 mV in **a** and 10 mV in **b** and **c**.

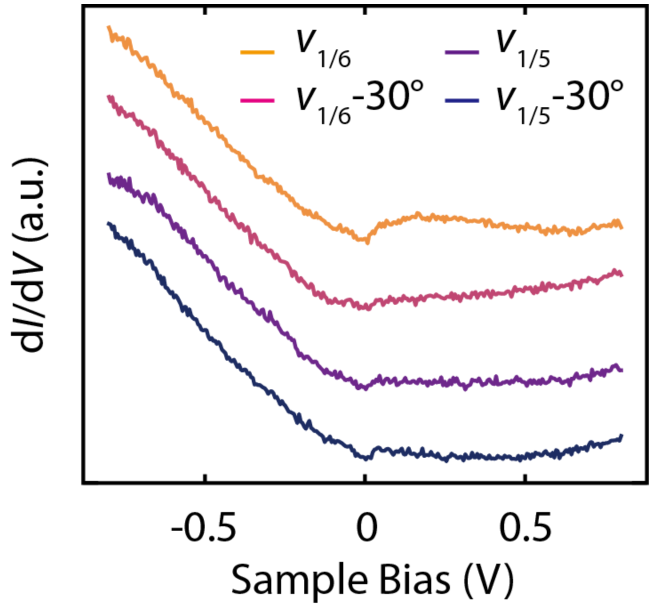

Supplementary Figure 6. STS spectra measured on different borophene phases, showing metallic behavior in all cases with subtle differences at positive bias.
